# Integrating hepatitis B virus, hepatitis C virus and human immunodeficiency virus screening for migrants from endemic countries into travel-related and sexual health care in Amsterdam, the Netherlands

**DOI:** 10.3389/fpubh.2025.1636918

**Published:** 2025-09-02

**Authors:** Ellen Generaal, Yara Bachour, Sara Klijzing, Akke Cornelissen, Roel Toering, Elske Hoornenborg, Eline Op de Coul, Nora Hamdiui, Annemarie Meiberg, Evelien Siedenburg, Maria Prins

**Affiliations:** ^1^Department of Infectious Diseases, Public Health Service of Amsterdam, Amsterdam, Netherlands; ^2^Amsterdam Institute for Immunology and Infectious diseases, Infectious Diseases, Amsterdam, Netherlands; ^3^Amsterdam Public Health, Global Health, Amsterdam, Netherlands; ^4^National Institute for Public Health and the Environment, Centre for Infectious Disease Control, Bilthoven, Netherlands; ^5^Department of Infectious Diseases, Amsterdam UMC Location University of Amsterdam, Amsterdam, Netherlands

**Keywords:** hepatitis B, hepatitis C, HIV, virus diseases, screening, migrants

## Abstract

**Introduction:**

The Health Council of the Netherlands has recommended screening for hepatitis B virus (HBV), hepatitis C virus (HCV) and human immunodeficiency virus (HIV) for migrants born in countries endemic for these infections. We investigated the operational feasibility, test uptake and diagnostic outcomes of integrated HBV/HCV/HIV screening into routine care at the Public Health Service (PHS) of Amsterdam.

**Methods:**

In 2022–2023, visitors born in endemic countries (≥ 18 years) were offered free HBV, HCV and HIV testing at the Travel and Vaccination Center (TVC) of the PHS. In 2021–2022, 1,172 visitors born in an endemic country for HCV (≥ 16 years) were offered free HCV testing at the Center for Sexual Health (CSH), alongside standard free HBV/HIV testing. Countries considered endemic for HBV and HCV had a prevalence of ≥2.0% or ranked among the top-10 endemic countries in the Netherlands. The screening program was considered operationally feasible if inclusion began within six months and ≥25% (TVC) or ≥50% (CSH) of eligible visitors were included within one year. Positivity rate was considered ‘low’ for prevalence < 2.0%, and ‘high’ for prevalence ≥ 2.0%.

**Results:**

At the TVC, 298 visitors participated in HBV (*n* = 264), HCV (*n* = 293) and/or HIV (*n* = 290) testing. At the CSH 1,023 visitors underwent HCV testing. Inclusion targets were met. Test uptake at the CSH was 87%; data for TVC were unavailable. At the TVC, we identified five newly diagnosed chronic HBV cases (2.0, 95%CI = 0.6–4.4%) and no new cases for HCV or HIV. At the CSH, one newly diagnosed chronic HCV case was identified (0.1, 95%CI = 0.01–0.5%).

**Discussion:**

Integrating HBV, HCV and HIV screening into routine travel-related care and additional HCV screening into sexual health care is operationally feasible. HBV screening at the TVC showed a high yield, while HCV and HIV yields were low. Routine HBV screening should be further examined in similar settings for migrants from endemic countries, preferably alongside HCV and HIV testing.

## 1 Introduction

In the Netherlands, Hepatitis B virus (HBV), Hepatitis C virus (HCV), and human immunodeficiency virus (HIV) are mainly diagnosed among key populations, including migrants born in countries where these infections are endemic: ‘endemic countries’ (1, 2). If left untreated, chronic viral hepatitis B and C can lead to long-term complications such as liver cirrhosis and hepatocellular carcinoma (3), while untreated HIV leads to acquired immune deficiency syndrome (AIDS) (4). Early diagnoses and treatment are crucial, not only for improving health outcomes but also for preventing onward transmission of these infections.

The prevalence of HBV, HCV and HIV in the Dutch general population is, respectively, estimated at 0.34, 0.04, and 0.2% (1, 5, 6). However, a study nested in the population-based HELIUS cohort in Amsterdam showed a higher chronic HBV prevalence, particularly among residents born in Ghana (5.4% of *n* = 495) and Turkey (4.1% of *n* = 496), compared to Amsterdam residents born in the Netherlands (0.4% of *n* = 462), and many were unaware of their infection (2). A screening study conducted at a medical practice for undocumented and uninsured individuals in Amsterdam in 2018–2019 also showed higher prevalence rates among its visitors compared to the general population, with 2.5% (of *n* = 438) for chronic HBV, 0.7% (of *n* = 435) for chronic HCV, and 1.1% (of *n* = 439) for HIV (7).

In the Netherlands, migrants from endemic countries are not routinely screened for these infections, despite recommendations from the Dutch Health Council (8) and in the Dutch National Hepatitis plan (currently updated; (9)). In addition, HBV and HCV screening of migrants from endemic countries is also recommended in the national guidelines for general practitioners (GPs) (10). Ideally, screening of individuals at risk for HBV, HCV and HIV should be embedded within the GPs standard of care, as they serve the majority of the population. Persons with a non-Western migration background visit their GP approximately a median five to seven times a year, more often than native Dutch residents (11, 12). Moreover, past HCV screening programs at Dutch GP centers have proven successful (13). However, a qualitative study in 2021 showed that GPs in Amsterdam (the Netherlands) do not implement screening on a regular basis, mainly due to time constraints and other practical barriers, such as the lack of knowledge on whom to offer testing, the inability to register country of birth in the electronic patient record, and financial barriers for the patient, as testing costs are part of the health insurance’s compulsory deductible (14). Therefore, ‘opportunistic screening’ in other settings than primary care should be examined, meaning testing offered to individuals during routine healthcare visits or other encounters with healthcare services, rather than through a population-based approach.

Given that previous studies showed that screening programs for HBV, HCV, and HIV among migrants from endemic countries were feasible and that there was willingness to be tested (2, 7), we set up a pilot program integrating free of charge screening of HBV, HCV and HIV for visitors with a migration background into routine care in a public health care setting. This screening program is grounded in principles of public health equity, aiming to ensure that migrants have equal and non-coercive access to healthcare services (15). The aim of this study was to assess the operational feasibility, test uptake, and diagnostic outcomes of this free of charge HBV, HCV and HIV screening program, integrated in routine care visits at the Travel and Vaccination Center and the Center for Sexual Health of the Public Health Service of Amsterdam, targeting visitors born in countries endemic for these infections.

## 2 Methods

### 2.1 Study population and design

The cross-sectional study was conducted from June 7th 2021, to June 1st 2022 at the Center for Sexual Health (further referred to as: ‘CSH’) and from July 4th 2022, to June 30th, 2023 at the Travel and Vaccination Center (further referred to as: ‘TVC’) of the Public Health Service (PHS) of Amsterdam, the Netherlands. Visitors of the TVC were offered free of charge testing for HBV, HCV and HIV. At the CSH, visitors were offered free of charge testing only for HCV, since HBV and HIV testing was already part of routine health care for migrants from endemic countries (and men who have sex with men) at the Centers for Sexual Health in the Netherlands.

### 2.2 Inclusion and exclusion criteria

Visitors aged 16 years or older at the CSH and 18 years or older at the TVC, residing in the Netherlands, and originating from a country endemic for HBV (at TVC) and/or HCV (both centers), were eligible for inclusion. Included countries of birth were those with an estimated prevalence of HbsAg or HCV-RNA equal or higher to 2%, in accordance with recommendations from the National Institute for Public Health and the Environment (16) and in line with previous cost-effective analyses for HBV screening among migrants (17). As the country list with high HBV and HCV prevalence estimates included all HIV-endemic countries, no additional countries were added for HIV testing (18).

In addition, we included all foreign-born visitors originating from the top ten countries of origin with the highest expected number of infections in the Netherlands, even if their estimated prevalence was below 2%, including Suriname, Morocco, India, and Egypt (1). The expected number of HBV or HCV infected individuals for these countries was the highest due to the large populations from these countries residing in the Netherlands (1).

**Figure 1:**
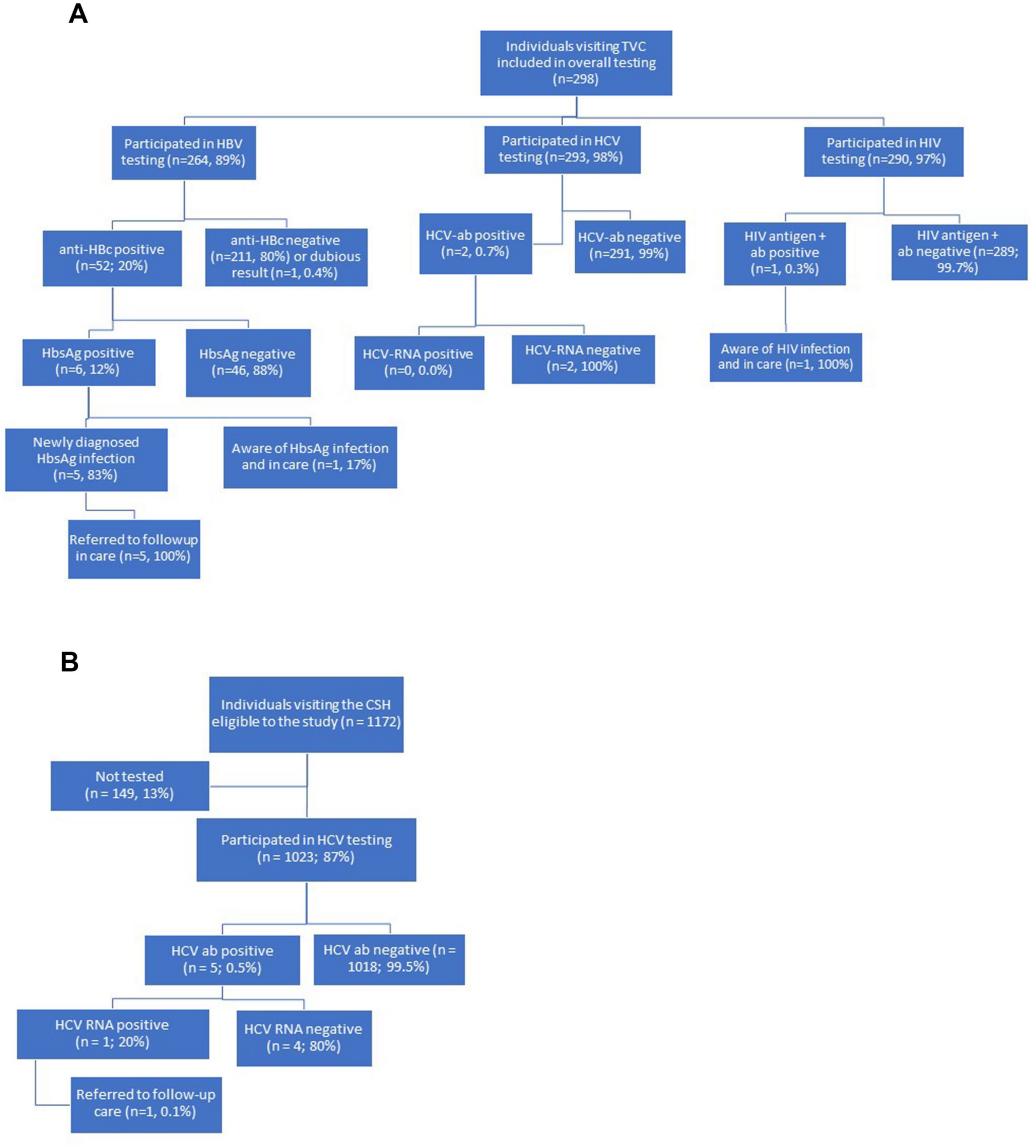
**(A)** Flowchart of recruitment, testing procedures, and linkage to care at the travel and vaccination center (TVC) of the public health service in Amsterdam, the Netherlands, from July 4th 2022 to June 30th 2023. **(B)** Flowchart of recruitment, testing procedures, and linkage to care at the center for sexual health (CSH) of the public health service of Amsterdam, the Netherlands, from June 7th 2021 to June 1st 2022.

Participants were also required to be able to understand the study information in Dutch or English. At the TVC, individuals were excluded from HBV testing if they had been (fully) vaccinated for HBV or were previously diagnosed with a cleared or occult infection (*n* = 31 of total *n* = 298). At the CSH, visitors living with HIV and those using HIV Pre-Exposure Prophylaxis (PrEP) were excluded from the study, as they already undergo regular testing for HBV, HCV and HIV as part of routine health care (data not shown). Visitors from the CSH who were already receiving care for HBV or HCV at a hospital were also excluded to prevent testing individuals who were already aware of their status. These excluded persons were not part of the participants eligible to the study (total eligible: *n* = 1,172; Figure 1B).

### 2.3 Study procedures

#### 2.3.1 Preparation and recruitment

Preparation of the studies involved minor adjustments to the electronic patient record (EPD) of the PHS, staff instructions at the centers, communication about testing procedures with the PHS laboratory, and writing a brochure on study procedures for the TVC visitors. At both test sites, participation of the visitor in the study was noted in the electronic patient record (EPD), at the TVC for each test separately. Demographic information had already been registered in the EPD at both centers as part of standard procedures. The number of eligible individuals who declined participation was available for the CSH, but not for the TVC. Reasons for non-participation were not formally registered at both centers, although most frequently mentioned reasons of refusal noticed by the staff of the TVC were a lack of perceived urgency to participate or a fear of needles.

#### 2.3.2 Screening procedures

##### 2.3.2.1 Travel and vaccination center (TVC)

Upon arrival at the TVC, visitors who met the inclusion criteria were informed about the study by a nurse and were handed over a brochure with the study information (Supplementary material 1) in the waiting room. In the consultation room, after consulting on (travel) vaccinations, the doctor inquired if the visitor was willing to participate in the screening program. Visitors were given the option to be tested for HBV, HCV and HIV, but they could freely choose which one(s) to test for. After obtaining verbal informed consent, a blood sample was obtained through venipuncture.

##### 2.3.2.2 Center for sexual health (CSH)

At the CSH, HCV testing was offered, in addition to the routine testing, by the nurse through verbal consent which was registered in the EPD. Blood collection was part of regular consultation.

#### 2.3.3 Follow-up procedures

Visitors who tested negative for all infections were either informed by letter (TVC) or online (CSH). If any of the test results was positive, medical personnel contacted the visitor who was then referred to a GP for linkage to care. The routine procedure also included contact tracing and partner notification in case of an acute infection.

### 2.4 Laboratory testing

Blood samples were sent to the Public health laboratory of the PHS of Amsterdam. Blood serum was screened for HBV, HCV and HIV infection by testing hepatitis B surface antigen (HBsAg), anti-Hepatitis B core (Anti-HBc) antibodies, anti-HCV antibodies, and HIV antigen and antibodies (LIAISON XL MUREX, DiaSorin, Italy). If the test result was positive for HBsAg or anti-HBc antibodies, further testing was performed including hepatitis B ‘e’ antigen (HBeAg), anti-Hepatitis B ‘e’ antibodies (anti-HBeAb), and anti-HBs antibodies. When anti-HCV-antibodies were present, HCV-RNA was determined on serum using the quantitative HCV-RNA test (Aptima HCV Quant Dx, Hologic). In the case of a positive HIV antigen or antibody result, confirmatory testing was performed using a Western blot assay (INNO-LIA™ HIV I/II Score, Auto-LiPA 48, Fujirebio), and an HIV-1 p24 antigen test (VIDAS HIV P24, mini VIDAS Blue, bioMérieux).

Participants with a positive anti-HBc antibody serology were defined as having been exposed to HBV. Based on the HBsAg test results, we further categorized these participants as either having a cleared or occult infection (HBsAg-negative) or having a chronic infection (HBsAg-positive). Similarly, individuals with positive anti-HCV antibody results were identified as having been exposed to HCV (i.e., HCV-exposed). Subsequently, based on HCV-RNA results they were further categorized as either having cleared the infection (undetectable HCV-RNA) or having an active infection (detectable HCV-RNA). Participants with confirmed positive tests for HIV antigen or antibodies were classified as HIV-positive.

### 2.5 Statistical analyses and outcome measures

Descriptive baseline characteristics were reported as medians with interquartile ranges (IQR) or as percentages with 95% confidence intervals (CI). Countries of birth were either reported individually or categorized into broader regions when fewer than ten individuals were recorded per country, using the classification of regions of birth by Statistics Netherlands (19). To determine the test uptake, we calculated the proportion of persons who agreed to participate among the total number of persons eligible for participation at the CSH. According to the European Centre for Disease Prevention and Control (ECDC), feasibility is defined as “the degree to which it is possible to implement an intervention in terms of time, money or other circumstances” (20). As this definition includes financial feasibility, among other aspects, we focus specifically on operational feasibility —the availability of personnel, equipment, processes, and organizational support to deliver the screening consistently (21). Operational feasibility was assessed by the time to implementation and the inclusion rate. The project was considered feasible if it could be launched within six months from project initiation to inclusion (excluding time for ethical approval), and if the target number of participants was enrolled within one year after the start of inclusion. The target inclusion rate was set at 50% of eligible visitors (≥ *n* = 1,000) at the CSH, which is in line with the participation rate found in a previous screening project among migrants from endemic countries at the tuberculosis (TB) facility of the PHS of Amsterdam (54%) (22). At the TVC, the target inclusion rate was set at 25% (≥ *n* = 250), which was calculated based on the number of eligible visitors in the year preceding the study. The lower target of 25% at the TVC reflects the limited availability of medical staff to recruit participants daily due to time constraints, although we acknowledge that this inclusion rate is at the lower end of what ECDC/EU studies report for community- and primary-care-based migrant screening (23).

For the diagnostic outcomes, we calculated the proportion of persons with a positive test for anti-HBc, HbsAg, HCV antibodies, HCV-RNA and HIV among those with a valid test result. Positivity rate was categorized as ‘high’ if the prevalence of infection was equal to or higher than 2%, and ‘low’ if below 2%, in line with WHO classifications that consider a prevalence of ≥2% as intermediate to high (24). In addition, we analyzed the proportion of persons unaware of infection among those with a positive test result, and the number of individuals referred to care. Success rate therapy was not recorded. In case of test positivity, we calculated the number needed to screen (NNS) as a crude estimate, to identify one person with an active infection by taking the inverse of the observed prevalence (1/prevalence) (25).

The 95% CIs were calculated using either the Clopper-Pearson or Jeffrey’s method, depending on whether the proportion of infected individuals was ≥0.01 or <0.01, respectively. For calculation of CIs of the NNS, we used the method by Altman et al. (26). Data were analyzed using SPSS statistics V.26.0.

## 3 Results

### 3.1 Operational feasibility, test uptake and description of the study population

#### 3.1.1 Travel and vaccination center (TVC)

At the TVC, the screening pilot was integrated into routine care within four months and target inclusion rate was met (*n* ≥ 250). A total of 298 visitors were tested for HBV, HCV and/or HIV. Participants had a median age of 38 years (IQR 31–57 years) and 55% (*n* = 165/298) were women (Table 1). Most participants at the TVC originated from Ghana (20%), Surinam (15%), Turkey (7%), or other countries within Africa (12%) or Asia (11%), as detailed in Table 1.

**Table 1:** Demographic variables of participants at the travel and vaccination center (TVC) and the center for sexual health (CSH) of the public health service of Amsterdam, the Netherlands.

		TVC (*n* = 298)	CSH (*n* = 1,023)
Inclusion period		July 4th 2022 – June 30th 2023	July 4th 2022 – June 30th 2023
Age in years (median, IQR)		38, IQR 31–57	28, IQR 23–34
Female sex, *n* (%)		165 (55%)	390 (38%)
Region or country of birth, *n* (%)			
Africa	Ghana	58 (20%)	0 (0%)
	Morocco	**	110 (11%)
	Other	37 (12%)	47 (5%)
Asia	China	15 (5%)	0 (0%)
	India	12 (4%)	55 (5%)
	Pakistan	11 (4%)	14 (1%)
	Syria	**	96 (9%)
	Other	32 (11%)	68 (7%)
Europe	Turkey	22 (7%)	0 (0%)
	Romania *	15 (5%)	107 (11%)
	Central Eastern Europe (CEE), other *	18 (6%)	60 (6%)
	Eastern-Europe #	14 (5%)	147 (14%)
	Other	5 (2%)	12 (1%)
Americas	South-America, Surinam	45 (15%)	307 (30%)
	South-America, other	2 (1%)	0 (0%)

*CEE countries are EU member states which were part of the former Eastern bloc; including Bulgaria, Czech Republic, Estonia, Hungary, Lithuania, Latvia, Poland, Romania, Slovenia, Slovakia^1^.

^#^Eastern-European countries include Russia and Ukraine for the CSH; and Russia, Ukraine, Belarus and Georgia for the TVC.

**The number of individuals born in these countries was smaller than 10, hence these countries were added to the subcategory ‘Other’.

CEE, Central and Eastern Europe; IQR, interquartile range; CSH, center for sexual health; TVC, travel and vaccination center.

^1^CEE countries (CEECs) | CBS.

#### 3.1.2 Center for Sexual Health (CSH)

The study was integrated into routine care at the CSH within two months and target inclusion rate was met (*n* ≥ 1,000). Of the 1,172 eligible visitors at the CSH, 1,023 (87%) were tested for HCV (Figure 1A) within the study period of one year. The remaining 149 visitors (13%) did not participate for unspecified reasons (Figure 1A). The 1,023 visitors had a median age of 28 years (IQR 23–34 years), and 38% (*n* = 390/1023) were women (Table 1). Most participants at the CSH originated from Surinam (30%), Morocco (11%), Romania (11%), or other Eastern-European countries (14%), including Russia and Ukraine. Additional details on countries and regions of birth are provided in Table 1.

### 3.2 Diagnostic outcomes: prevalence of HBV, HCV, and HIV infection

#### 3.2.1 Travel and Vaccination Center (TVC)

Of the 298 included visitors at the TVC, 264 (89%) were tested for HBV, 293 (98%) for HCV and 290 (97%) for HIV (Figure 1A). Of the 264 participants tested for HBV, 52 (20%; 95%CI = 15–25%) were positive for anti-HBc antibodies. Of these, 46 (88%; 95%CI = 77–96%) had a negative HbsAg result and were defined as having a cleared or occult infection, and 6 (12%; 95%CI = 4.4–23%) had detectable HbsAg (see Figure 1A). The overall HbsAg prevalence among participants was 2.4% (95%CI = 0.8–4.9%, *n* = 6/246; Table 2). All participants with HbsAg-positivity originated from the African region with five from Ghana and one from Somalia. Of the six participants with positive test results for HbsAg, five (83%; 95%CI = 36–99%) were unaware of their chronic HBV infection (Figure 1A) and they were referred to care. The one participant who was aware of the HbsAg infection was already receiving care at a clinic. The number needed to screen to detect one person with a chronic HBV infection was estimated at 41 (crude estimate; 95%CI = 19–111).

**Table 2:** Test results among participants at the travel and vaccination center (TVC) and the center for sexual health (CSH) of the public health service of Amsterdam, the Netherlands.

	TVC (*n* = 298^*^)	CSH (*n* = 1,023)
HBV		
Anti-HBc positivity, *n* (%, 95 CI)	52 (20%, 15–25%)	Not investigated
HbsAg positivity, *n* (%, 95CI)	6 (2.4%, 0.8–4.9%)	Not investigated
Newly diagnosed HbsAg infections, *n* (%, 95CI)	5 (2.0%, 0.6–4.4%)	Not investigated
HCV		
HCV-ab positivity, *n* (%, 95CI)	2 (0.7%, 0.1–2.2%)	5 (0.5%, 0.2–1.1%)
Overall HCV-RNA positivity, *n* (%, 95CI)	0 (0.0%, 0.0–0.9%)	1 (0.1%, 0.01–0.5%)
HIV		
HIV antigen + ab positivity, *n* (%, 95CI)	1 (0.3%, 0.04–1.6%)	Not investigated

* Total number of individuals tested at the TVC: *n* = 246 for anti-HBc and HbsAg, *n* = 293 for HCV-ab, and *n* = 290 for HIV.

Anti-HBc, hepatitis B core antibodies; CI, confidence interval; HbsAg, hepatitis B surface antigen; HCV-ab, hepatitis C antibodies; HIV, human immunodeficiency virus; CSH, center for sexual health; TVC, travel and vaccination center.

Of 293 participants tested for HCV at the TVC, two individuals (0.7, 95%CI = 0.1–2.2%), one born in Ghana and one in Surinam, were positive for HCV-antibodies (Table 2). Both had undetectable HCV-RNA (overall prevalence = 0.0%; 95%CI = 0.0–0.9%; Table 2). Among the 290 individuals tested for HIV, one tested positive (0.3, 95%CI = 0.04–1.6%) (Table 2). This participant was previously diagnosed and in care (Figure 1A).

#### 3.2.2 Center for Sexual Health (CSH)

Of the 1,023 included visitors at the CSH, five tested positive for HCV antibodies (0.5, 95%CI = 0.2–1.1%; Table 2). In one of these five participants (20, 95%CI = 0.5–72%), HCV-RNA was detected, indicating an active infection (Figure 1B). Of the total sample (*n* = 1,023), HCV-RNA prevalence was 0.1% (95%CI = 0.01–0.5%). Four participants had either spontaneously cleared HCV-infection or were successfully treated for HCV (two from Surinam and two from Russia). The participant with an active HCV infection, born in Russia, was unaware of the infection and was referred to the hospital for follow-up care (Figure 1B).

## 4 Discussion

In the pilot HBV, HCV and HIV screening program integrated into routine care at the Travel and Vaccination Center (TVC) and the Center for Sexual Health (CSH) of the Public Health Service of Amsterdam, we investigated the uptake, operational feasibility, and diagnostic outcomes among visitors originating from endemic countries.

The test uptake for HCV -the only test offered at the CSH as HBV and HIV testing was already part of routine health care for this group- was high at CSH (87%). The fact that testing was free of charge and blood samples were already obtained for routine sexual health care likely contributed to the high uptake. At both sites, the screening project was easily implemented within two to four months and the targeted number of participants was successfully enrolled.

At the TVC, six out of 246 participants (2.4%; 95%CI = 0.8–4.9%) had a chronic HBV-infection, with five unaware of their status. No new HCV or HIV infections were identified, only one previously diagnosed HIV-infection. At the CSH, one new active HCV-infection (0.1%; 95%CI = 0.01–0.5%) and five resolved HCV-infections (0.5%; 95%CI = 0.2–1.1%) were found, with four unaware of their prior infection. These prevalence estimates for chronic HBV and active HCV are generally in line with previous studies, but are lower for HIV (7). A 2013–2015 screening study among migrants at the Amsterdam PHS TB facility, which also integrated HBV, HCV and HIV testing into routine care, found a lower chronic HBV prevalence (0.39%; 1/256; 95% CI = 0.07–2.18), a higher chronic HCV prevalence (0.39%; 1/256; 95% CI = 0.07–2.18), and no HIV infections - all previously diagnosed (22). The observed differences in findings might be explained by inclusion of different migrant populations. Our study demonstrates that each public health site serves a different migrant population, varying by country of birth, age, and gender. This highlights the importance of implementing opportunistic screening programs across diverse public healthcare settings, with adaptations tailored to the specific needs and context of each setting.

In parallel with our study, two other pilot studies were conducted in 2023 by the PHS of Limburg South and Groningen, targeting HBV and HCV among migrants from a specific endemic country (27). While non-integrated programs are often time-limited and labor-intensive, they have the advantage of reaching populations that do not frequently attend care facilities. In Limburg South, Syrian migrants were identified via municipal registries and invited by post for point-of-care testing at the PHS (27). The study achieved the inclusion target (269 tested of 250 aimed, 32% participation rate), but no new HBV and HCV infections were found (27). In Groningen, first-generation Somali migrants were recruited through an asylum center, but the reach was low -only 29 individuals were tested (target: 250), and again, no HBV and HCV infections were detected (personal communication). These outcomes underscore the need for alternative approaches to identify undiagnosed cases, such as opportunistic screening embedded in routine care, as shown in our study. Our broader inclusion of endemic countries may also enhance test uptake and diagnostic yield compared to the other pilots.

Opportunistic screening programs integrated into routine care, as highlighted in a systematic review (28), present three major advantages: no need for active recruitment, the use of familiar healthcare settings, and the ability to maintain low-cost, ongoing screening and follow-up. According to the ECDC (29) and WHO (30), integrated screening is essential to enhance early detection, improve linkage to care, and ensure timely treatment, particularly among underserved populations such as migrants. While our study focused on the public health care setting, integrated testing has also been examined in other countries and health care settings, such as primary care (31), TB testing facilities (22, 32), hospital emergency departments (33, 34) and medical services in prisons (35). These studies generally show high test yields but mixed results regarding linkage to care. Notably, a systematic review of qualitative studies among migrants residing in EU/EAA-countries showed that trust, cultural sensitivity, and communication skills of healthcare providers have implications for linkage to care (36). These factors should be considered when designing future interventions (36) – ideally through co-development with migrant populations to overcome barriers faced in accessing services (31). These screening programs should be integrated into comprehensive approaches that address the full spectrum of migrants’ health needs and vulnerabilities, with dedicated efforts to support this goal (37).

For a low-endemic country as the Netherlands, combined HBV and HCV screening has been proven cost-effective for migrants born in the top-10 endemic countries (with the highest number of infected HBV and HCV cases), in particular for opportunistic facilities within routine care and community-based settings (38). Although not directly supported by our pilot study, we suggest to combine HBV testing with testing for HCV and HIV. This combined approach will identify more undetected infections, may potentially clarify transmission pathways and prevention measures, and may increase screening uptake (39). Moreover, ‘triple testing’ for HBV, HCV and HIV aligns with the WHO’s recommendations, is only slightly more expensive and serves as a low-cost interim step toward the WHO’s targets (40). However, more research is needed on cost-effectiveness, long-term linkage to care outcomes and the impact on onward transmission of these combined screening programs (24, 41). These screening programs for those born in endemic countries should be integrated across various existing health care facilities, along with outreach activities, to prevent major complications and mortality by these infections and transmission to other individuals (42). This approach aims to support to the achievement of the Sustainable Development Goal to eliminate these epidemics by 2030 (29, 40). Guaranteeing migrants access to appropriate healthcare aligns with public health priorities and the human rights principle of non-discrimination, and should be a core responsibility of host countries (43).

Our study has several limitations. First, the exact number of eligible visitors at the TVC and reasons for non-participation were not carefully recorded due to time constraints, limiting conclusions on test uptake at TVC and reasons for refusal at both centers. Second, the small number of positive cases (especially for HCV and HIV) limits the precision and power of prevalence estimates. Third, the low number of infections and lack of data on behavior associated with infection precluded the ability to examine potential determinants of infection. Fourth, while we observed a high test uptake at CSH (87%), which suggests the screening program was implementable within the given context, we did not directly assess participant perceptions nor clinical outcomes. Therefore, no conclusions can be drawn regarding the acceptability and effectiveness of the screening program. Fifth, we cannot rule out potential selection bias in our study, since visitors with a positive attitude towards testing (and venipuncture at TVC) may be more willing to participate. Lastly, we studied migrant groups visiting two centers of the PHS in Amsterdam and our findings may not be generalizable to other migrant contexts or national programs. This highlights the need to expand screening programs to community-based settings to reach more diverse migrant populations at risk of HBV, HCV or HIV. Screening programs that compare different recruitment and screening strategies are needed to examine the effectiveness of these approaches (28). In addition, future screening programs should consider to use self-testing or self-sampling, as these methods might help to reduce barriers such as stigma and time constraints, thereby promoting more equitable access to screening, provided that proper education and guidance are given to those being tested (44).

One of the key strengths of our study is its setting within the Public Health Service (PHS) of Amsterdam, a densely populated city (931,748 residents in 2023) (45) with a large first-generation migrant population (36% in 2023) (46). This enabled us to include a broad spectrum of visitors from numerous countries where HBV, HCV, and HIV are prevalent. Additionally, a substantial proportion of eligible visitors were successfully enrolled in the study within the expected time frame. We attribute this achievement to the fact that testing was offered free-of charge, the effort required from participants was minimal (as blood samples were already collected at the CSH), and experienced medical personnel were available, trained to interact with visitors from diverse ethnic backgrounds (47). Other favorable factors identified in our study, which are also relevant to integrated testing programs more broadly, include the selection of migrants from a diverse range of endemic countries (varying by site), the convenience of receiving services at a location they were already visiting, (often for other medical purposes, such as testing or vaccination) and the rapid, operationally feasible, and affordable rollout of the screening pilot.

## 5 Conclusion

This study, conducted at the Public Health Service of Amsterdam, investigated the test uptake, operational feasibility, and diagnostic outcomes of integrating HBV, HCV, and HIV screening into routine travel-related and sexual health centers for visitors originating from endemic countries. The approach proved to be feasible for service providers to implement, achieved high participation rates, and identified several previously undiagnosed infections. The findings underscore the benefits of embedding a case-finding program within health care settings. Additional research—such as studies in rural areas and on cost-effectiveness—along with adequate funding and political support, is essential for the structural implementation of such programs in public health care settings.

## Data Availability

The raw data supporting the conclusions of this article will be made available by the authors, without undue reservation.
