# Supplementary material for: Integrating hepatitis B virus, hepatitis C virus and human immunodeficiency virus screening for migrants from endemic countries into travel-related and sexual health care in Amsterdam, the Netherlands
Source: Front Public Health. 2025 Sep 2;13:1636918. doi: 10.3389/fpubh.2025.1636918 (PMC12439341; doi:10.3389/fpubh.2025.1636918)
Supplement: Supplementary file 1 [file Table_1.DOCX]

**
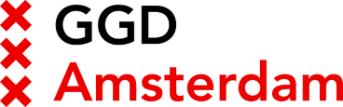
Supplement 1: brochure with information about the HBV, HCV, HIV screening
project at the Travel Center**

Testing for hepatitis B, hepatitis C and HIV in visitors of the Travel Center

1. Why is testing important?

- Hepatitis B and C are diseases of the liver. These diseases can cause liver damage and liver cancer.
- HIV is the virus that causes AIDS. AIDS makes you very sick.
- You can have these diseases for a long time without noticing.
- It is important to find the diseases in time by testing. If you start taking medication on time, you can usually live a long and healthy life with the disease.
- There is a vaccine against hepatitis B. There is no vaccine against hepatitis C or HIV.

2. Who can get tested?

-
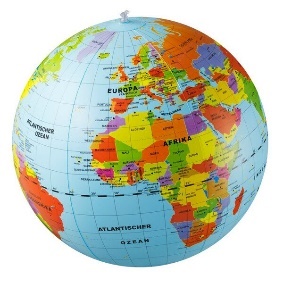
Hepatitis B, C and HIV are uncommon in the Netherlands, but much more common in other countries.
- We have a list of these countries (± 80) and can see if your

country of birth is included.

- If the diseases are common in your country of birth, you can get tested for these diseases.

3. Do you want to participate in the study?

- The GGD offers free tests for the diseases. This is done by taking two tubes of blood.
- You can choose which diseases you want to be tested for.
- The nurse or doctor will ask you if you want to participate. He/she can also answer questions.

4. After testing:

- If you do not have the diseases, you will receive a letter at home.
- If you do have one of the diseases, you will be called.
  Then the GGD will tell you that you can come back or ask you to go to the
  hospital. They will check if you need medication.

5. Purpose of the study

The GGD wants to know whether it makes sense to offer travellers tests for these diseases more often. In this way they can improve the care they provide.

The GGD is working together with the RIVM in this investigation. The GGD shares anonymous figures with the RIVM. The GGD is obliged to keep your name and other personal information secret.
